# Research Progress in Preparation, Properties and Applications of Biomimetic Organic-Inorganic Composites with “Brick-and-Mortar” Structure

**DOI:** 10.3390/ma16114094

**Published:** 2023-05-31

**Authors:** Feng Liu, Hongyu Yang, Xiaming Feng

**Affiliations:** College of Materials Science and Engineering, Chongqing University, Chongqing 400044, China; 20210901002@cqu.edu.cn

**Keywords:** bio-inspired, organic–inorganic composite, “brick-and-mortar” structure, research progress

## Abstract

Inspired by nature, materials scientists have been exploring and designing various biomimetic materials. Among them, composite materials with brick-and-mortar-like structure synthesized from organic and inorganic materials (BMOIs) have attracted increasing attention from scholars. These materials have the advantages of high strength, excellent flame retardancy, and good designability, which can meet the requirements of various fields for materials and have extremely high research value. Despite the increasing interest in and applications of this type of structural material, there is still a dearth of comprehensive reviews, leaving the scientific community with a limited understanding of its properties and applications. In this paper, we review the preparation, interface interaction, and research progress of BMOIs, and propose possible future development directions for this class of materials.

## 1. Introduction

**Figure 1:**
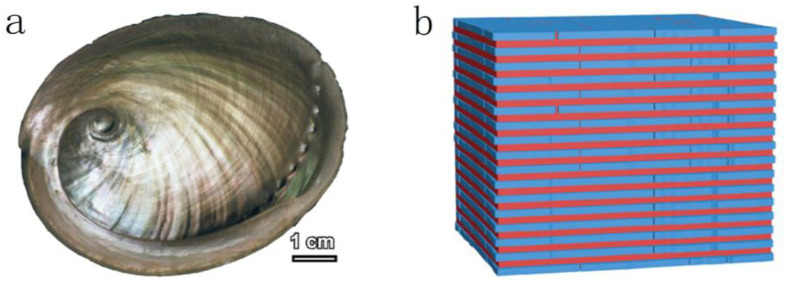
(**a**) Image of the inner, iridescent part of the shell of Haliotis laevigata [3]; (**b**) schematic of BMOI [4].

Over the past few centuries of biological evolution, nature has produced numerous astonishing natural materials. This has inspired the design of composite materials, such as biomimetic organic–inorganic composites with a “brick-and-mortar” structure (BMOIs), which take inspiration from nacre. Nacre (Figure 1a) is composed of 95% brittle aragonite platelets and 5% soft biological macromolecules arranged in a highly ordered “brick-and-mortar” structure (Figure 1b) that reveals exceptional mechanical properties [1,2,3,4]. Research on nacre structure [5,6,7] has demonstrated the critical role of its “brick-and-mortar” structure in conferring its superior properties. Xu et al. [8] used atomic force microscopy to study the behavior of biopolymer chains in the nacre and have found that the biopolymer matrix can self-reinforce during deformation. Researchers found that the biopolymer matrix in the nacre has the ability to self-strengthen during deformation. The nacreous structure, the “brick-and-mortar” structure, is able to deflect and inhibit crack propagation, resulting in enhanced toughness and efficient dissipation of mechanical energy [9,10,11,12,13]. Therefore, BMOIs have emerged as a type of composite material with excellent properties and multiple applications, prepared using inorganic materials as “bricks” and organic materials as “mortar” through specific methods.

As early as 1989, Sarikaya et al. [14] proposed the “brick-and-mortar” structure, analyzed its architecture, and subsequently utilized it to design and synthesize synthetically laminated materials. Over the past few decades, corresponding research on BMOIs based on the “brick-and-mortar” structure has become increasingly refined through both theoretical and practical developments. Nowadays, a diverse range of raw materials are utilized, with commonly used inorganic materials including montmorillonite (MMT) and graphene oxide (GO), which possess excellent barrier properties and design flexibility. As early as 2007, Lin et al. [15] developed a “brick-and-mortar”-structured nanocomposite material by incorporating polymers into the interlayer of MMT. In 2011, Putz et al. [16] proposed a method of preparing “brick-and-mortar” structured paper using GO. Moreover, researchers have employed various synthetic methods, such as layer-by-layer assembly (LBL), vacuum filtration (VF), co-extrusion, and freeze casting (FC). Therefore, BMOIs, with their diverse selection of raw materials and synthetic approaches, have enjoyed great expansion in terms of its research and application scope.

In recent years, BMOIs have gained increasing attention and found wide applications in fields such as biomedicine, flame retardancy, and intelligent materials. However, in the existing reviews on relevant topics, most of them include BMOIs as a part of the review without specific targeting. Some research focuses on the mechanical behavior [17] and shear transfer efficiency [18] of nacre. Some consider it as a part of a bionic application review (such as acoustic materials [19], structural materials [20], thermal conductive materials [21], nanochannels [22], energy absorbing materials [23], fiber composites [24,25] and gels [26]). Some incorporate BMOIs as part of certain materials (such as graphene [27,28]) or preparation methods (such as freezing [29,30,31] and self-assembly [32]) reviews, while others only review certain applications of BMOIs (such as oil-repellent and water-repellent materials [33] and biomedical materials [34]). The existing reviews mostly explain certain aspects of BMOIs and cannot provide a comprehensive understanding of them. Therefore, by providing a detailed description of the preparation and applications of BMOIs, we aim to provide a comprehensive understanding of BMOIs and inspire researchers from different fields to study them.

## 2. Raw Materials

It is well known that the properties of composite materials are largely influenced by their constituent materials. Common inorganic materials in BMOIs include montmorillonite, graphene oxide and MXene, while organic materials include polyvinyl alcohol, cellulose and its derivatives. Other less commonly used raw materials include chitosan and calcium carbonate. A list of raw materials is presented in Table 1.

### 2.1. Inorganic Raw Material

#### 2.1.1. Montmorillonite

**Figure 2:**
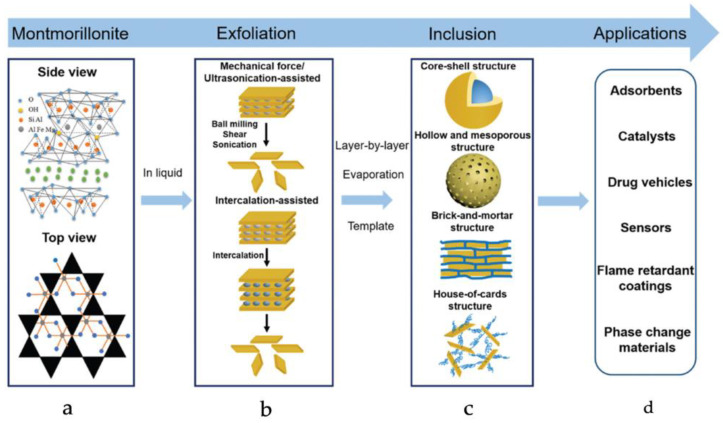
Schematic diagram of (**a**) structure, (**b**) exfoliation, (**c**) inclusion and (**d**) application of montmorillonite [50].

Montmorillonite (MMT) is an excellent inorganic material that, when introduced into materials, can significantly improve their corrosion resistance, fire resistance, antibacterial activity, adsorption, and other properties. It is commonly used as an adsorbent, catalyst, flame retardant, and carrier. Its structure, exfoliation, preparation, and applications are shown in Figure 2 [49,50]. Due to its rich interface functional groups, MMT can form strong interface interactions with organic materials and is widely studied as an inorganic material in BMOIs.

**Figure 3:**
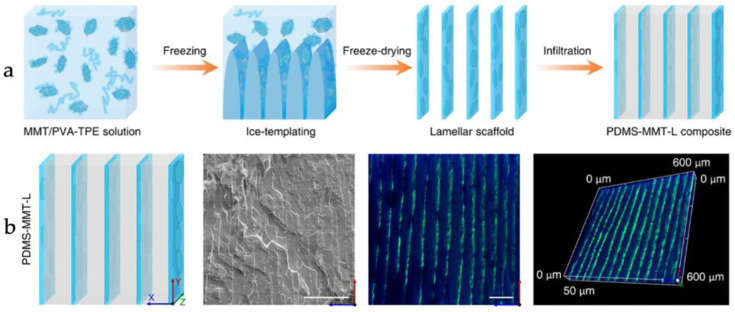
(**a**) Diagram of the PDMS-MMT-L manufacturing method; (**b**) schematic illustration (from left to right is SEM image, CFM image at the XY plane, as well as the three-dimensional reconstruction) [35].

Peng et al. [35] employed the ice-templating technique to fabricate a nanocomposite material, referred to as PDMS-MMT-L, by introducing MMT and polydimethylsiloxane, resulting in a “brick-and-mortar” structure, as depicted in Figure 3a. The PDMS-MMT-L exhibited a substantial improvement in Young’s modulus and toughness, by 23-fold and 12-fold, respectively, when compared to pure polydimethylsiloxane. The toughening mechanism of the material, involving crack deflection and bridging, was validated by in situ crack tracking and three-dimensional reconstruction, utilizing the pearl-activated PDMS-MMT-L nanocomposite material and confocal fluorescence microscopy (CFM), assisted by the aggregation-induced emission (AlE) (Figure 3b).

**Figure 4:**
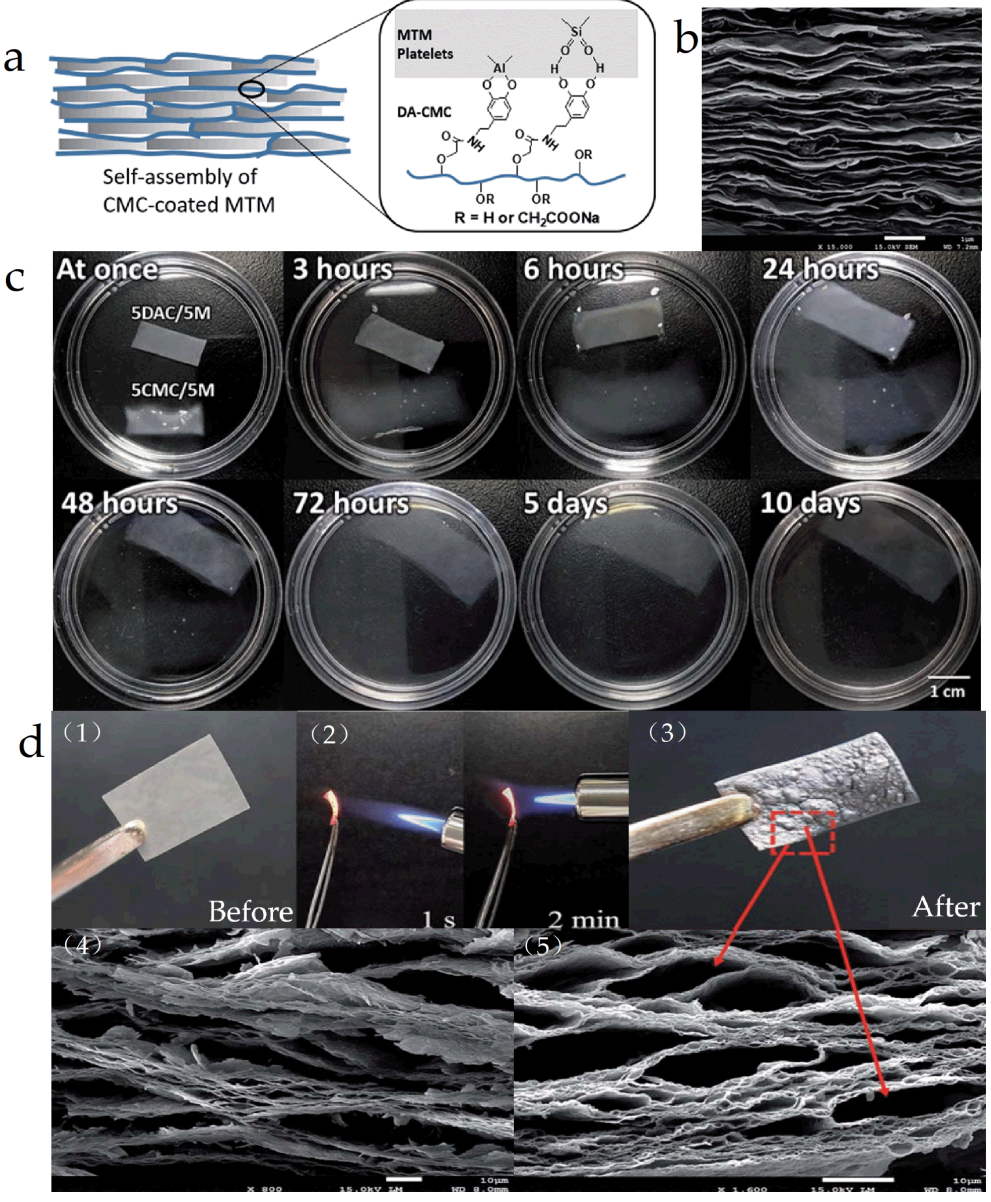
(**a**) The schematic of DAC/MTM; (**b**) the SEM image of DAC/MTM; (**c**) water stability test diagram of the DAC/MTM; (**d**) diagram of DAC/MTM exposed to gas combustion process and SEM after combustion, (1) DAC/MTM before combustion, (2) gas combustion process (3) DAC/MTM after combustion, (4, 5) SEM after combustion of the DAC/MTM in (3) [36].

Guo et al. [36] coupled multifunctional carboxymethyl cellulose to dopamine and assembled it with montmorillonite using a vacuum filtration-assisted method to prepare a “brick-and-mortar structured” material (DAC/MTM, Figure 4a,b). The material was characterized using X-ray photoelectron spectroscopy (XPS), nuclear magnetic resonance NMR, and other tests, and it was found that it could persist in water for more than ten days (good wet-state stability) (Figure 4c), high tensile strength (162.0 MPa), and elastic modulus (8.7 GPa) when the relative humidity reached 90%. In addition, thermal analysis and horizontal flame testing demonstrated that the material also had excellent fire resistance (Figure 4d).

Rahid et al. [37] used a layer-by-layer deposition technique to form a film composed of polyethyleneimine (PEI), montmorillonite, and oligomeric siloxane. The film showed a nearly 65 GPa reduced modulus due to strong electrostatic and hydrogen bonding. Additionally, the material exhibited high hardness and chemical resistance, making it durable over time.

#### 2.1.2. Graphene Oxide

Graphene oxide (GO) is a two-dimensional nanomaterial with controllable size and excellent mechanical, electrical, chemical, and optical properties. It also has excellent modifiability, as its plethora of epoxy functional groups and hydroxyls on the outside allow it to react very easily with other polymers to improve the mechanical properties of materials [51]. In addition, GO has excellent electrical and thermal conductivity, making it a valuable material for applications in electronics, sensors, and energy storage devices. Its optical properties also make it an attractive material for applications in optoelectronics and photonics. This indicates that GO is well-suited as a “brick” for preparing multifunctional BMOIs with mechanical properties.

**Figure 5:**
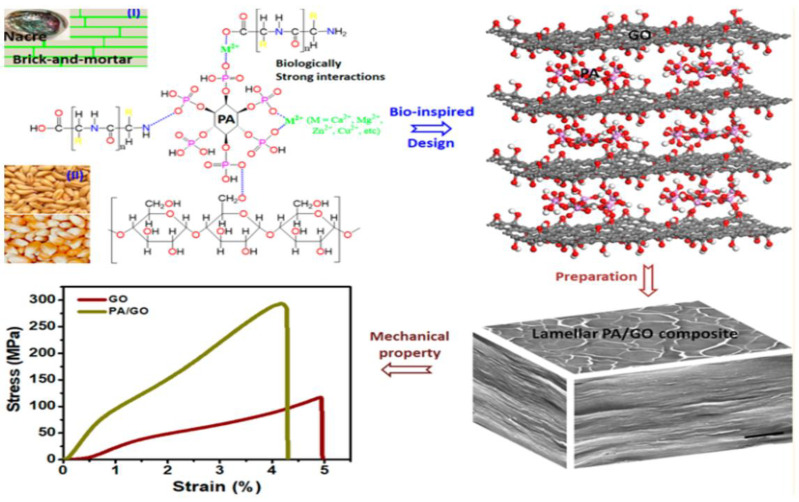
Schematic diagram of PA/GO preparation and comparison of tensile test results between PA/GO and GO [38].

Chen et al. [38] combined the natural chelating agent phytic acid (PA) with GO, and they were closely linked by hydrogen bonds to form a “brick-and-mortar” structure (Figure 5). Compared with pure GO, the hardness, ultimate strength, maximum Young’s modulus, toughness, and strain energy release rate of the nacre-bio-based composite material (PA/GO) increased by 41.0%, 124.1%, 134.7%, 118.5%, and 150.6%, respectively.

**Figure 6:**
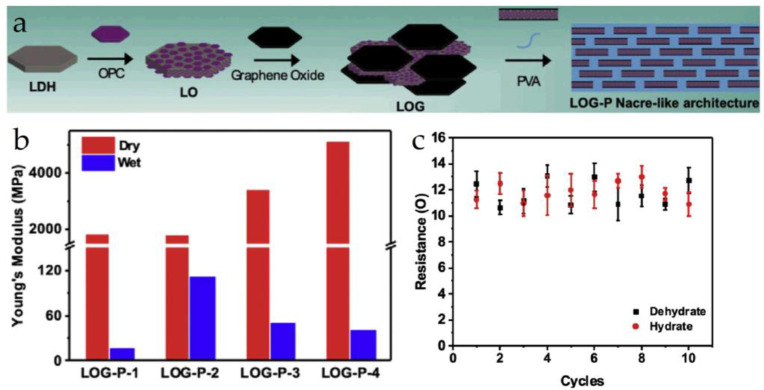
(**a**) Composite plot of LOG-P; (**b**) graph of the resulting Young’s modulus of LOG-P; (**c**) cyclic resistance measurements of Ag electrodes in hydrated and dehydrated states [39].

Yu et al. [39] synthesized a composite material substrate named LOG-P by intercalating oligomeric proanthocyanidins (OPC) into Ca-Al layered double hydroxide (Ca-Al-LDH) and GO, and then crosslinked it with 2D polyvinyl alcohol (PVA) to construct a “brick-and-mortar” structure, as shown in Figure 6a. The Young’s modulus of LOG-P in the dehydrated state is about 5 GPa, while it decreases by approximately 99% in the hydrated state, as shown in Figure 6b. Ag circuits were integrated on LOG-P using 3D printing technology to study the electrical stability of microelectronic structures and evaluate the feasibility of LOG-P as a human body implant, as shown in Figure 6c.

**Figure 7:**
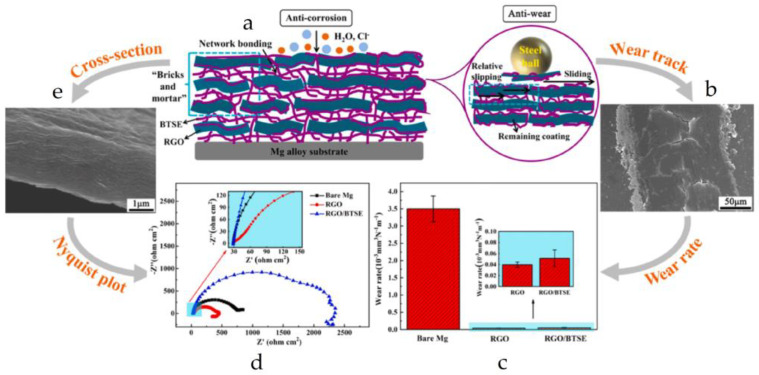
(**a**) Schematic diagram for the corrosion/wear resistance mechanism of RGO/BTSE coating; (**b**) SEM image of RGO/BTSE coating after wear; (**c**) COF value of samples (bare Mg, RGO, RGO/BTSE) under the calculated wear rate; (**d**) results of electrochemical measurement; (**e**) sross-sectional SEM images of coated RGO/BTSE samples [40].

Chu et al. [40] successfully grafted a reduced graphene oxide (RGO)/bis (diethoxyphosphoryl) ethane (BTSE) coating onto the surface of magnesium alloy substrate (Figure 7a), while inhibiting corrosion and wear behavior. The coating exhibited a well-arranged biomimetic “bricks and mortar” structure connected by a chemical network, composed of Si-O-C bonds between RGO flakes and Si-O-Si bonds originating from BTSE self-crosslinking reaction. The reduction in porosity and defects made the RGO/BTSE coating highly continuous and intact, and the corrosion rate of the coating was reduced by an order of magnitude compared to the bare Mg substrate and RGO-coated samples due to barrier effect (Figure 7d). Furthermore, the wear resistance of the substrate was found to be improved, as indicated in Figure 7b,e, which was attributed to the directional lubrication effect of reduced graphene oxide flakes. The composite coating also exhibited excellent wear resistance, providing protection against frictional wear of the substrate. The wear rate decreased from 3.5 × 10^−3^ mm^3^ N^−1^ for bare magnesium alloy to 5.13 × 10^−5^ mm^3^ N^−1^ m^−1^ for RGO/BTSE coated samples (Figure 7c). The interlayer sliding between RGO flakes effectively reduced the friction resistance, although the addition of BTSE weakly increased its shear resistance.

#### 2.1.3. MXene

**Figure 8:**
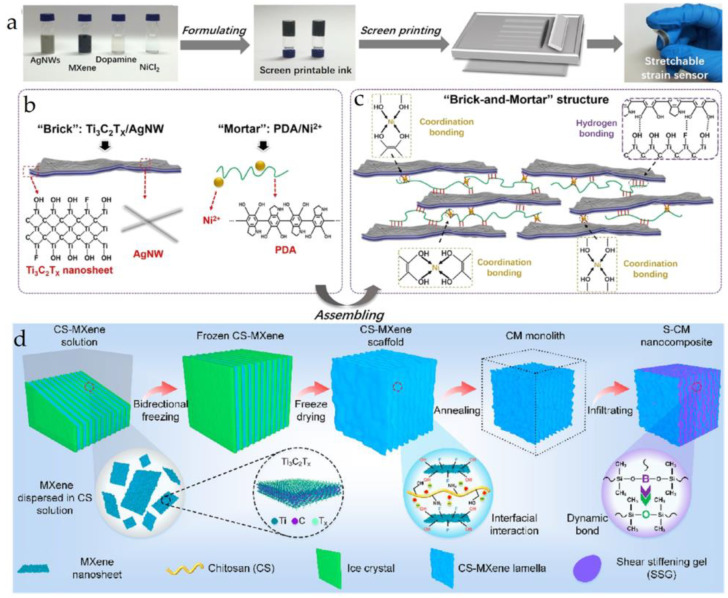
(**a**) Flow chart of the preparation of Ti_3_C_2_Tx-AgNW-PDA/Ni^2+^; (**b**) schematic representation of the structure of “brick” and “mortar”; (**c**) schematic representation of “brick and structure” [41]; (**d**) flow chart of the preparation of chitosan/MXene [42].

MXene is a new type of 2D transition metal carbide/nitride material with atomic layer thickness, large electrochemically active surface energy, and excellent mechanical properties. Its general formula can be represented as M_n+1_X_n_T_x_, where M represents an early transition metal, X represents carbon and/or nitrogen, and T_x_ represents functional groups attached to the surface of MXene during etching. Its structure is similar to that of graphene. MXene has made exciting achievements in research fields such as hydrogen storage, supercapacitors, and electromagnetic shielding. Shi et al. [41] used hydrophilic 2D titanium carbide (Ti_3_C_2_T_x_) MXene nanosheets and highly conductive 1D silver nanowires (AgNWs) as “bricks”, poly(dopamine) (PDA)/Ni^2+^ as “mortar”, and developed a strain sensor with a “brick-and-mortar structure”, which has ultra-high sensitivity, high strength, and toughness, as shown in the flow chart in Figure 8a and the “brick-and-mortar structure” in Figure 8b,c. Liu et al. [42] used freeze-drying to construct a chitosan/MXene layered structure skeleton embedded in a polyborosiloxane matrix to create a “brick-and-mortar structure” composite material with favorable anti-impact and electromagnetic interference (EMI) shielding performance. The preparation process and structure schematic are shown in Figure 8d. The material can effectively attenuate 85.9–92.8% of the impact force and has a highly efficient EMI shielding effect of 47.2–71.8 dB.

At times, multiple inorganic materials are used at the same time. Chen et al. [43] used GO, MMT, and wood auto-hydrolysates (WH) to prepare a nanocomposite thin film. The film was strengthened by the synergistic effect of hydrogen bonding and covalent bonding between the three components, resulting in excellent mechanical and fire-resistant properties.

### 2.2. Organic Raw Material

#### 2.2.1. Polyvinyl Alcohol (PVA)

**Figure 9:**
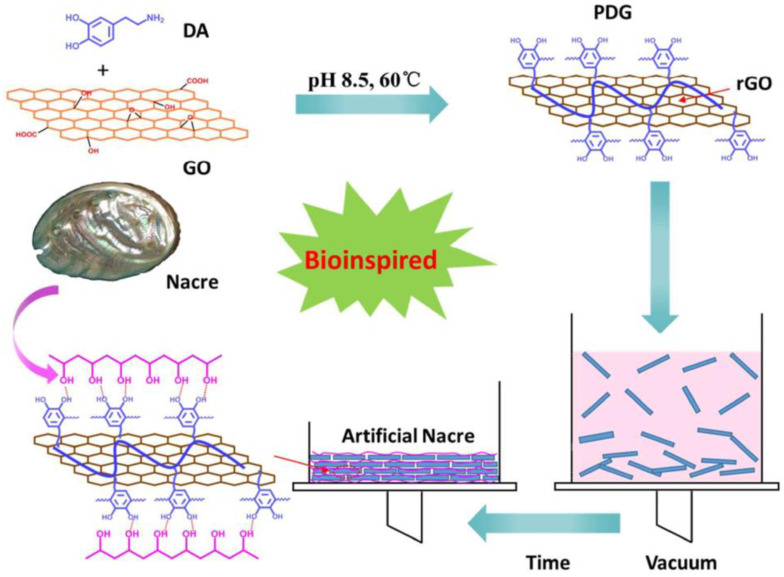
Scheme for the preparation of the PDG–PVA [45].

Polyvinyl alcohol (PVA) is widely used as a coating material, packaging film, and in the textile industry due to its excellent film-forming and barrier properties. Moreover, its biocompatibility and ability to form hydrogels have made it a subject of extensive research for biomedical applications such as drug delivery and tissue engineering. Therefore, PVA is considered an excellent organic material with diverse potential applications [44,45]. Kou et al. [44] used reduced graphene oxide (CRG) as “bricks” and PVA as “mortar” to prepare a coating (CRG-PVA) with a “brick-and-mortar structure” and outstanding mechanical properties. Kilometers-long fibers were then prepared using continuous wet-spinning assembly technology. The tensile strength of the fiber increased with the PVA content, reaching a maximum of 200 MPa, surpassing other nacre-inspired materials. It was found that the tensile strength of the fibers was not related to the molecular weight of PVA, as molecular motion is confined to the nanochannels between adjacent CRG layers. Wang et al. [45] used polydopamine-wrapped reduced graphene oxide (PDG) as “bricks” and PVA as “mortar” to prepare an artificial nacre nanocomposite material using vacuum-assisted filtration self-assembly technology, as shown in Figure 9. The material’s tensile strength, fracture elongation, and toughness were 327 ± 19.3 MPa, 8 ± 0.2%, and 13.0 ± 0.7 MJ/m^3^, respectively, which is significantly higher than nacre, mainly due to the “brick-and-mortar” structure of PDG and PVA and the hydrogen bonds between them.

#### 2.2.2. Cellulose and Its Derivatives

**Figure 10:**
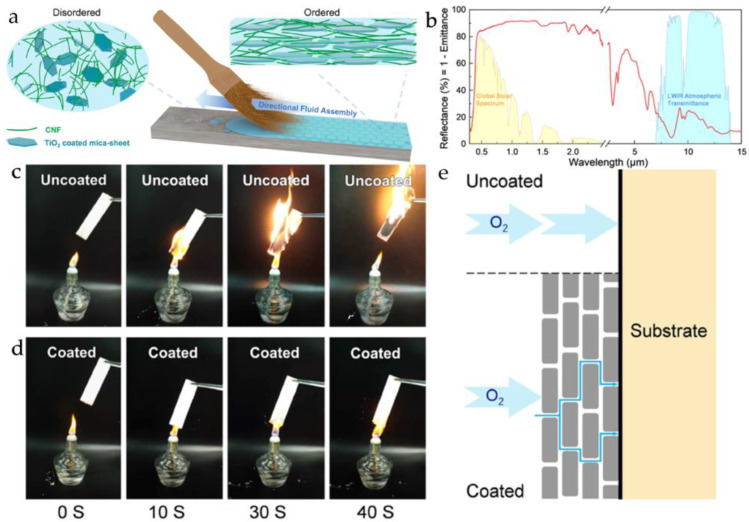
(**a**) Preparation of CN- TiO_2_-mica; (**b**) infrared reflectance performance test of CN-TiO_2_-mica; (**c**,**d**) fire test of balsa wood and balsa wood with CN-TiO_2_-mica coating; (**e**) schematic diagram of the principle of high fire-retardant performance of the nacre-inspired coating [46].

The primary skeleton of cellulose is synthesized by plants through photosynthesis, making it an abundant and renewable resource [52]. Cellulose exhibits biodegradability and good chemical modification capabilities, enabling it to meet the design requirements for intermolecular interactions and environmental friendliness for biomaterials of interest. Guan et al. [46] utilized cellulose nanofiber and TiO_2_-coated mica microplatelets (TiO_2_-mica) to prepare an environmentally friendly water-based material (CN-TiO_2_-mica) that combines energy-saving cooling and fire-resistant properties. By employing the “directional fluid assembly” method to construct a “brick-and-mortar structure” (Figure 10a), high infrared reflectivity (about 90%, Figure 10b) can be achieved. The fire resistance can be improved by prolonging the path of oxygen entry into the material (Figure 10c–e). Qiu et al. [47] utilized nanofibrillar cellulose and hydroxyl functionalized black phosphorus to produce a high mechanical strength and highly efficient flame-retardant film. Xu et al. [48] used carboxymethyl cellulose (a cellulose derivative) and nanoclay platelets to prepare a high-tensile-strength, high-transparency, and fire-resistant film.

## 3. Strategies

### 3.1. Layer-By-Layer Assembly

**Figure 11:**
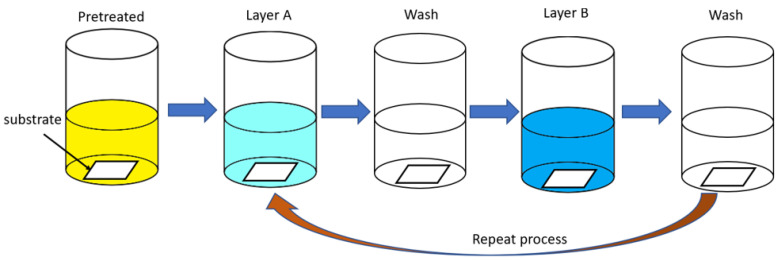
Preparation process of Al_2_O_3_/GO-PVA [55,56].

The layer-by-layer assembly (LBL) technique, originally proposed by Iler in 1966 [53], has since evolved to include the assembly of multilayer thin films in three dimensions, significantly expanding its applications. This technique is a bottom-up processing method that enables the accurate sort of nanoscale components based on layer-by-layer adsorption, resulting in materials with a clear nanostructure. As such, layer-by-layer assembly has become a commonly used method for creating BMOIs [54]. The layer-by-layer assembly process involves substrate pretreatment, layer deposition driven by a driving force, cleaning between layers to stabilize the layer structure, and cyclic repetition of the process [55,56], as shown in Figure 11.

**Figure 12:**
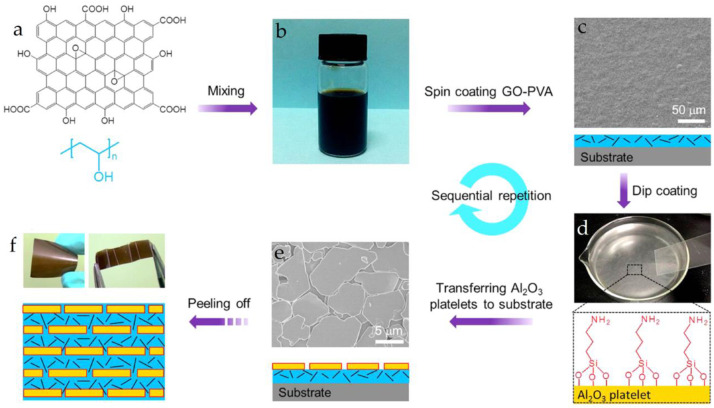
The preparation process of Al_2_O_3_/GO-PVA. (**a**) GO and PVA. (**b**) mixing of GO and PVA. (**c**) GO-PVA coating and its SEM image. (**d**) assembling silane-modified Al_2_O_3_ microplatelets into monolayer at air−water interface. (**e**) transferring the assembled Al_2_O_3_ monolayer onto GO−PVA layer. (**f**) the obtained flexible, foldable artificial nacre after sequential repetition [57].

In a study by Wang and colleagues [57], aluminum oxide/GO-PVA (Al_2_O_3_/GO-PVA) nanosheets were used to create artificial pearls. The process involved adding 3-aminopropyltriethoxysilane to a solution of water and methanol, stirring until it was fully hydrolyzed, and then adding Al_2_O_3_ platelets and sonicated them. After stirring at 40 °C for 30 min, they obtained a suspension that was washed with ethanol (Figure 12). Using this suspension and a GO/PVA dispersion, they alternately dried the films on a glass substrate to produce a thin film, which had superior strength (143 ± 13 MPa) and toughness (9.2 ± 2.7 MJ/m^3^) compared to nacre. Moreover, the tensile strength of the Al_2_O_3_/GO-PVA film was 2.8 times that of the Al_2_O_3_/PVA film, and the toughness was about 6 times that of the GO-PVA film. These results suggest that the three-component composite material is effective in achieving a balance between strength and toughness.

### 3.2. Vacuum Filtration

**Figure 13:**
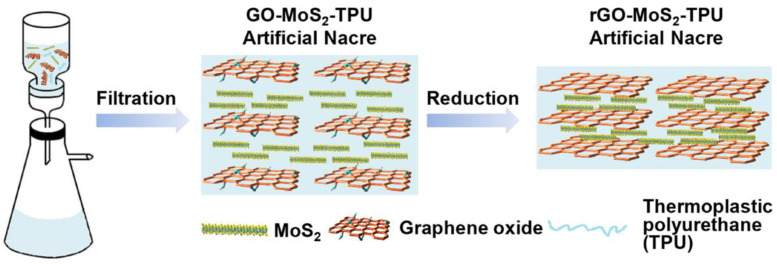
Diagram of the synthesis process of GO-MoS_2_-TPU [59].

Vacuum filtration (VF) [58], also referred to as papermaking, is a classical mechanical assembly method that uses a vacuum pump to pass a solution through a filter, ultimately resulting in solid film formation. The primary benefit of this approach is its expandability, which enables it to be used for large-scale manufacturing. However, assembling bulk materials can be time consuming. Wan et al. [59] based their work on GO–molybdenum disulfide (MoS_2_)–thermoplastic polyurethane (TPU). They added the MoS_2_ and TPU solutions to the GO solution, and after stirring and ultrasound, a suspension was obtained. The artificial nacre layer was obtained by vacuum-assisted filtration and drying, and then immersed in an HI solution for reduction. The final product was obtained by washing with ethanol, as shown in Figure 13. This film not only has high toughness (3.8 times that of natural nacre layer) and strength (1.7 times that of natural pearl layer), it also has high conductivity, making it highly promising in applications such as artificial muscles, tissue engineering, and flexible supercapacitor electrodes.

### 3.3. Freeze Casting

**Figure 14:**
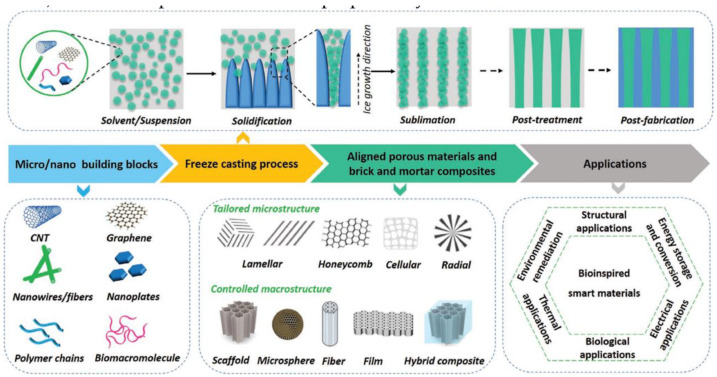
Schematic diagram of principle, structure application of FC [31].

In seawater, when the water contains impurities, ice crystals can form a mechanism that excludes impurities into the crevices of the ice [60]. Inspired by this appearance, the technology of freeze casting (FC) has emerged. Freeze casting is a technology that enables the formation of ordered, layered porous materials and is currently a commonly used technique for preparing biomimetic “brick-and-mortar” structure [61]. Shao et al. [31] introduced the principles of FC technology in different directions and analyzed the relationship between the process and the structure. As shown in Figure 14, the basic principles of FC technology are first described. FC involves the controlled solidification of solutions, suspensions, sols, or gels, followed by sublimation of the solvent under reduced pressure, and then densification through post-processing. During the controlled solidification process, phase separation occurs as the solvent solidifies, and the resulting solid phase (usually ice) serves as a template.

Munch et al. [62] combined alumina with poly(methyl methacrylate) to form a super-tough, template-structured hybrid ceramic material, with a processing flowchart. The hybrid ceramic material has toughness similar to aluminum alloy. In the past, most FC techniques used isotropic and unidirectional FC, but the nucleation direction of isotropic FC is uncontrollable, and unidirectional FC produces only a near-distance regular multilayer structure. To improve FC technology and establish long-range, regular layered structures, bidirectional FC was developed [63]. Zhao et al. [64] utilized an improved bidirectional FC method and then assembled a GO/PVA composite film using uniaxial extrusion and chemical reduction. Consequently, a composite film was developed that not only had high strength (150.9 MPa), but also toughness (8.50 MJ/m^3^) and high elongation (10.44%), which is superior to materials prepared by other methods.

### 3.4. Other Methods

Co-extrusion is a large-scale processing technique utilized to produce “brick-and-mortar” structures comprising ceramics with metallic toughness. In this method, the viscosity of the polymer is reduced by extruding raw material rods through a heated spinneret, resulting in the formation of long fibers collected online by the extrusion head. The co-extruded long fibers, such as NiO-coated Al_2_O_3_, are cut into short segments and subjected to transverse deformation by heating and pressing in a mold, followed by bonding to construct a “brick-and-mortar” structure [65,66,67].

3D printing has recently been used in constructing nacre-inspired “brick-and-mortar” structures with complex 3D shapes [68]. Specifically, inorganic materials are arranged in an orderly manner during the 3D printing process, assisted by the application of external fields (electric field, force, electric, magnetic field, etc.). Zhang et al. [69] used sodium alginate (SA) and GO to synthesize a multiresponsive, “brick-and-mortar”-structured sheet that exhibits rapid responses to multiple stimuli (water, heat, light). The 3D-printed pearls are lightweight and exhibit mechanical properties comparable to natural nacre. 3D printing technology has the advantage of producing pearl-like materials with complex three-dimensional shapes that are difficult to obtain by conventional methods, without compromising their scalability and shape-forming ability. However, controlling the microstructure without compromising the expandability and shape-forming ability is a challenge for 3D printing technology.

Advantages and disadvantages (Table 2): FC allows for fine control over layer thickness and mimics some of the nanoscale structural features of nacre. However, it cannot provide fine control over platelet size distribution, especially at scales less than 1 μm. In contrast, LBL assembly technology is suitable for the production of high-precision, regular nanocomposites, resulting in well-defined nanostructures. This not only allows for highly layered and ultra-thin composite materials, but also typically results in strong interfacial bonding and mechanical strength due to close contact between layers. However, due to its multi-step nature, LBL technology is very time consuming and labor intensive. Vacuum filtration is energy efficient, but the synthesized materials often have low density and high porosity, leading to poor mechanical properties.

## 4. Interfacial Forces

The interface interaction between organic and inorganic materials is an important factor affecting the performance of BMOIs. To adjust the mechanical properties of BMOIs based on the requirements of materials properties and processes, different interface interactions can be constructed. Common interface interactions include covalent bonds, hydrogen bonds, ionic bonds, and so on. Song et al. [51] investigated the interfacial design and toughening mechanism of GO-based biomimetic mineralized composites (BMOIs). They used GO with different sizes as “bricks”, PVA as “mortar”, and carbon nanotubes (CNTs) as interfacial reinforcement to prepare four composite materials with the same structure but different interface distributions. By comparing the results of tensile tests with finite element simulation results, they found that the bonding strength and stress level between the hard phase interfaces were the key factors determining the overall mechanical properties of the material/organization.

### 4.1. Non-Covalent Bonds

#### 4.1.1. Hydrogen Bonds

Hydrogen bonds are among the most frequently encountered and essential intermolecular or intramolecular interactions in molecules [70]. Despite being weak, they can have a profound impact on the aggregation state of a substance by modifying its physical properties, shape, and structure [71]. Dikin et al. [72] prepared ordered layered structures of graphene oxide thin films by filtration method. Due to the hydrogen bonding between GO and water, the GO paper exhibited high mechanical properties with a tensile strength of 133 MPa and a Young’s modulus of 32 GPa.

Kang et al. [73] simulated the structural features of natural nacre by combining (GO) and gelatin (GG) biopolymer and revealed the mechanism of comprehensive mechanical properties. The material was induced to self-assemble into orderly arrangements of nacre-like films through the formation of coordination bonds, ionic bonds, and hydrogen bonds by vacuum filtration, resulting in a strong and tough BMOI with a fracture strength of 88.7 MPa, a fracture strain of 0.84%, a tensile modulus of 25.4 GPa, and good biocompatibility.

#### 4.1.2. Ionic Bonds

**Figure 15:**
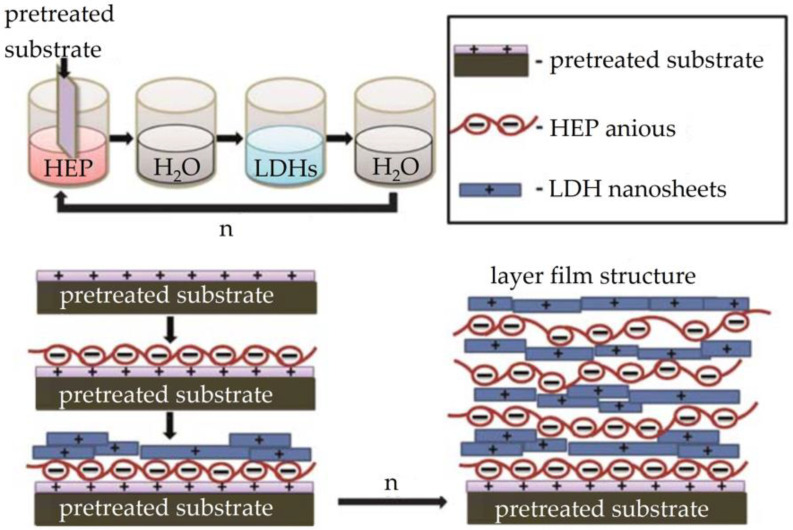
Preparation and interfacial force diagram of (LDH/HEP)_n_ [74].

Shu et al. [74] used a bottom-up LBL deposition technique to prepare ultra-thin films (LDH/HEP)_n_ with strong electrostatic and hydrogen bonding at the interface of nacre-like heparin (HEP) and layered double hydroxides (LDH), as shown in Figure 15. The HEP/LDH ultra-thin films were densely stacked together, forming a clear “brick-and-mortar” structure, demonstrating significant mechanical property enhancement and good biocompatibility. Compared with previously reported polymer-LDH hybrid films, the film modulus was significantly increased (23 GPa).

Liang et al. [75] prepared a high-performance BMOI with excellent UV-blocking ability, semi-transparency, and mechanical properties. They used a fast and green vacuum filtration method, selecting polyglutamic acid (PGA) as the “mortar” and positively charged NiAl-NO_3_ LDH nanosheets as the “brick” to prepare the PGA/LDH film through strong electrostatic interaction. Experimental results including XRD, FT-IR, and tensile tests indicated that the artificial pearl had a layered “brick-and-mortar” structure, excellent strength (93.5 MPa), and flexibility (easy to bend and fold).

#### 4.1.3. π-π Bonds

**Figure 16:**
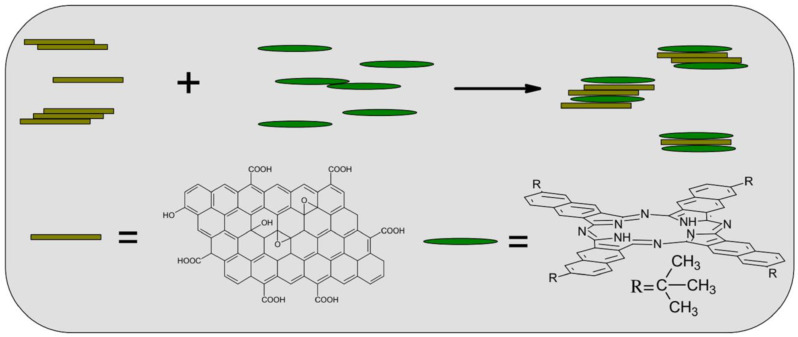
Schematic diagram of preparation of GO-NPc [77].

Graphene, with its SP^2^ hybridized structure, has strong stability due to π-π bonding. Similarly, BMOI with strong stability can be constructed through π-π interactions of GO. Zhang et al. [76] synthesized poly (acrylic acid) and 3-aminophenylboronic acid to form PAPBx, and then used room-temperature casting and drying method to prepare a GO hydrogel with a thickness of 10~12 μm by enhancing the π-π interactions between PAPBx and GO nanosheets. The gel prepared at a PAPBx concentration of 4% showed excellent tensile strength, fracture strain, and toughness, which were 382 ± 12 MPa, 4.31 ± 0.08%, and 7.50 ± 0.40 MJ m^−3^, respectively. Zhang et al. [77] used a π-π stacking supramolecular method to fix phthalocyanine derivative-2,11,20,29-tetra-tert-butyl-2,3-naphthalocyanine (NPc) on GO to form a layered GO-NPc hybrid, as shown in Figure 16. Adsorption and fluorescence spectroscopy analyses confirmed the existence of π-π interactions between GO and NPC, making the GO stable under environmental conditions. Fluorescence and photo-response measurements also showed that this hybrid material could be used as an effective photoelectric conversion material for photoelectric applications.

### 4.2. Covalent Bonds

**Figure 17:**
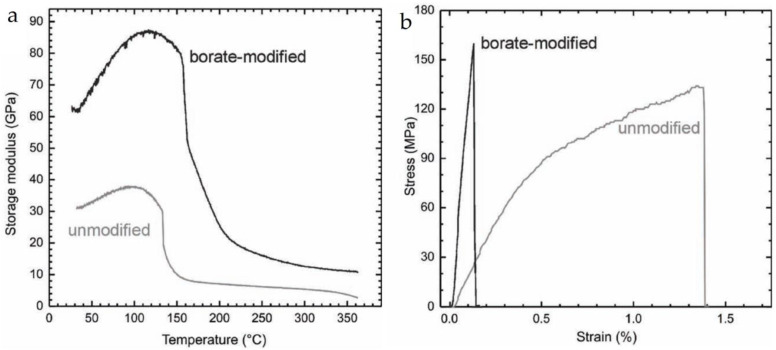
(**a**) Storage modulus of SPI-Gly rGO film; (**b**) stress–strain of SPI-Gly rGO film [80].

Although the aforementioned non-covalent bonds can significantly improve the mechanical properties, they are easily disrupted in solution, salt, and pH values [78]. To expand the application of BMOIs, it is necessary to use stronger covalent bonds to prepare BMOIs. Common covalent bonds include amide bonds and boronic ester bonds. Liu et al. [79] utilized a vacuum-evaporation self-assembly technique to insert graphene oxide (GO) nanosheets into a Soy protein isolate (SPI)-glycerin (Gly) matrix, developing a biomimetic composite material film that solves the problems of poor mechanical performance and water resistance of SPI. The composite material (SPI-Gly) constructed a “brick-and-mortar” structure through hydrogen bonds and amide bonds, resulting in improved tensile strength and modulus. After chemical reduction, SPI-Gly rGO was obtained, with further improvements in tensile strength (50.03 ± 2.67 MPa) and modulus (1.7 ± 0.07 GPa). Meanwhile, the SPI-Gly rGO film can maintain its original shape in water for up to one year, as shown in Figure 17. An et al. [80] formed a rigid-layered GO–borate BMOI by borate salt cross-linking. The results showed that the strength of the oxidized graphene film increased by nearly 25% to 160 ± 18 MPa with the addition of 0.94 wt% borate salt, and the stiffness increased by more than 255%.

## 5. Properties and Application

### 5.1. Light Weight and High Strength

**Figure 18:**
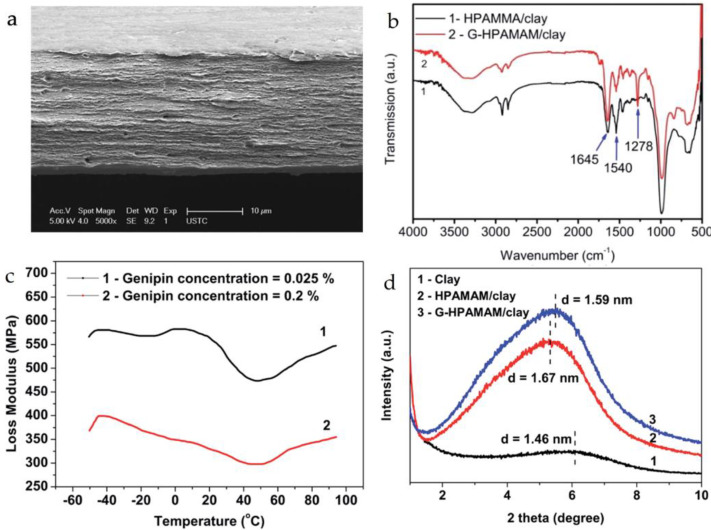
(**a**) SEM images of HPAMAM/nanoclay composite (5000×); (**b**) FT-IR of G-HPAMAM/nanoclay composite film; (**c**) loss modulus of G-HPAMAM/nanoclay composites; (**d**) SAXD patterns of G-HPAMAM/nanoclay composites [81].

The adoption of a “brick-and-mortar” architecture has been demonstrated to provide exceptional mechanical robustness to biomimetic materials composed of intercalated nanosheets. Remarkably, several of these materials have even outperformed natural nacre in terms of mechanical strength, meeting the high demands of materials for fields such as construction, aviation, and biomedical engineering that require high strength and low weight. Hao and colleagues [81] used genipin crosslinking, hyperbranched poly (amido amine) (HPAMAM), and clay nanosheets to prepare a tough biomimetic composite membrane (G-HPAMAM/nanoclay) through SEM observation with highly ordered structures (Figure 18a), similar to those of natural nacre and nacre-like composite materials. FT-IR testing (Figure 18b) revealed a peak at 1278 cm^−1^, indicating the presence of genipin in the pearl-like composite material, and the vibration peaks near 1540 cm^−1^ were weakened, while those near 1645 cm^−1^ were relatively enhanced. These results indicate that an amide connection was formed between genipin and HPAMAM. As shown in Figure 18c, the biomimetic composite membrane had two relaxation peaks in the loss modulus at −40 °C and 7 °C, respectively, with the peak at −40 °C indicating strong mobility of HPAMAM molecules, and the broader peak at 7 °C suggesting that the relaxation time distribution of HPAMAM/nanoclay was wide, but could be inhibited by an increase in genipin concentration. As shown in Figure 18d, the diffraction peaks of G-HPAMAM/nanoclay were shifted more significantly compared with those of the uncrosslinked composite material, indicating a reduction in the interlayer spacing of clay nanosheets. The interaction between HPAMAM and clay nanosheets was stronger after crosslinking because the close binding of HPAMAM molecules resulted in a shorter distance between the clay nanosheets. This suggests that the biomimetic composite membrane has good flexibility. Finally, the fracture toughness of the biomimetic composite membrane was as high as 5.03 MJ/m^3^, and the mechanical strength was 152.9 MPa, far exceeding that of nacre.

### 5.2. Flame Retardant

In recent years, research on “brick-and-mortar” structures in the field of flame retardancy has rapidly gained popularity. The flame-retardant effect of these structures is mainly achieved by forming a barrier on the surface, isolating the substrate from contact with air, and simultaneously inhibiting heat conduction. The materials can be applied in fields such as construction and electronics that are prone to fire due to its high flame retardancy. By reducing the probability of fire occurrence, it can help to minimize casualties and property damage caused by fires.

Carosio et al. [82] prepared “brick-and-mortar”-structure composite materials using transparent cellulose nanofiber/clay nanocomposites, which can be applied as a fire retardant coating for wood. This coating not only enhances the fire resistance of the wood, but also has a certain degree of optical transparency. The flame-retardant performance was evaluated using cone calorimetry. When exposed to a typical 35 kW/m^2^ heat flux, the ignition time of the coated wood sample was improved to about 4.5 min, and the maximum average heat release rate was reduced by 46%, the total heat release during combustion was reduced by 33%, thereby significantly reducing the potential fire hazard of the wood structure.

**Figure 19:**
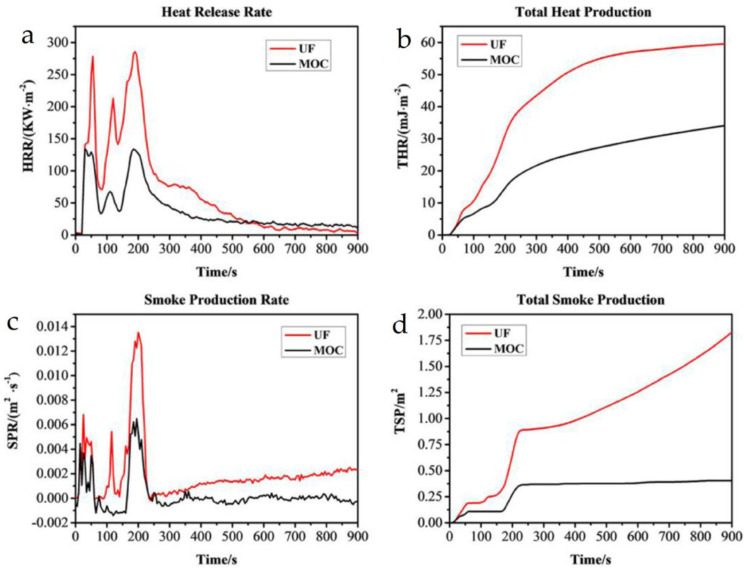
Plot of the results of the cone calorimetric test of the LVL. (**a**) HRR, (**b**) THR, (**c**) SPR and (**d**) TSP in cone calorimetry test [83].

Meanwhile, Sun et al. [83] used chloromagnesite cement as a substitute for traditional adhesives and D-gluconic acid sodium salt as a waterproof modifier in the preparation of laminated veneer lumber (LVL) to produce a “brick-and-mortar”-structure LVL with high strength and flame retardancy. The fracture strength of the LVL was about 101 MPa, which is equivalent to that of LVL produced with currently available commercial adhesives. Cone calorimetry testing showed a significant decrease in heat release rate (HRR), total heat release (THR), smoke production rate (SPR), and total smoke production (TSP), as shown in Figure 19.

**Figure 20:**
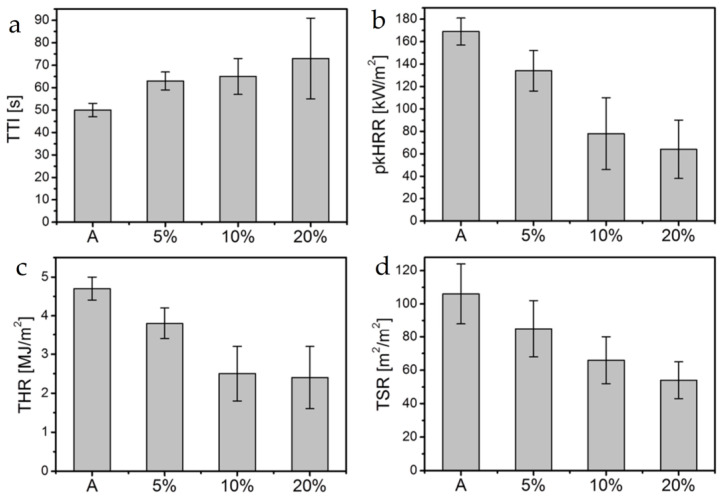
Plot of the results of the cone calorimetric test of the composite material (**a**) TTI, (**b**)pkHRR, (**c**)THR and (**d**)TSR in cone calorimetry test [84].

Carosio et al. [84] obtained compressed chitosan/montmorillonite complexes by directly mixing solutions/suspensions of opposite charges, deposited multilayer coatings using the LBL deposition technique, and then coated the coatings onto acrylic fabrics using a simple doctor-blade coating technique. The structure of the composite was studied by field emission scanning electron microscopy (FESEM), and it was found that oriented clay nanoplatelets were continuously bonded together by the chitosan matrix. Increasing the coating loading to above 10% improved the uniformity of the surface coverage. The coating with a loading of over 10% endowed the fabric with self-extinguishing ability in flame retardancy tests. Cone calorimetry tests showed that the ignition time was prolonged (up to 46%), the heat release rate was significantly reduced (the peak heat release rate and total heat release rate were up to −62% and −49%, respectively), and the total smoke release was also reduced (up to −49%), as shown in Figure 20.

**Figure 21:**
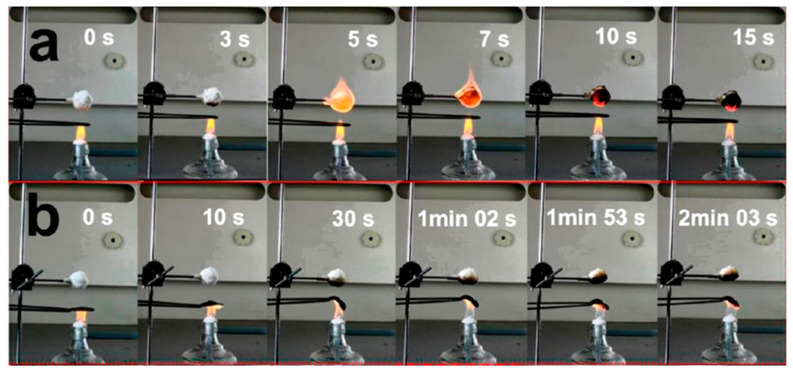
Fire test: (**a**) the combustion result of a cotton ball; (**b**) combustion results of cotton ball protected by BP−OH40/NFC [47].

Zhong et al. [85] developed a material with ultra-high strength and stiffness using injection molding and a combination of organophilic ultrathin γ-Al(OH)_3_ (O-gibbsite) single-crystal nanoplatelets, all-hydrocarbon composites, and extended-chain ultrahigh-molecular-weight polyethylene (UHMWPE). The material is constructed with O-gibbsite as bricks and UHMWPE as mortar. The tensile strength of the material reached 200 MPa, and the notched Izod impact strength was 28 kJ/m^2^, significantly better than other commercial HDPE and hydrocarbon matrix (All-PE) materials. Despite the high combustibility of hydrocarbons, the material still exhibited good flame retardancy. Inspired by the natural pearl layer, Qiu et al. [47] proposed a bio-inspired nanocomposite material composed of nanofiber cellulose (NFC) and a few layers of hydroxyl-functionalized black phosphorus (BP-OH) using a vacuum-assisted filtration self-assembly process. The two-dimensional (2D) BP-OH dispersed well in the one-dimensional (1D) NFC and had a strong interface hydrogen bond, resulting in the composite film having excellent tensile strength and elongation at break, reaching 214.0 MPa and 23.8%, respectively. The material also exhibited high heat and fire resistance, as shown in Figure 21.

**Figure 22:**
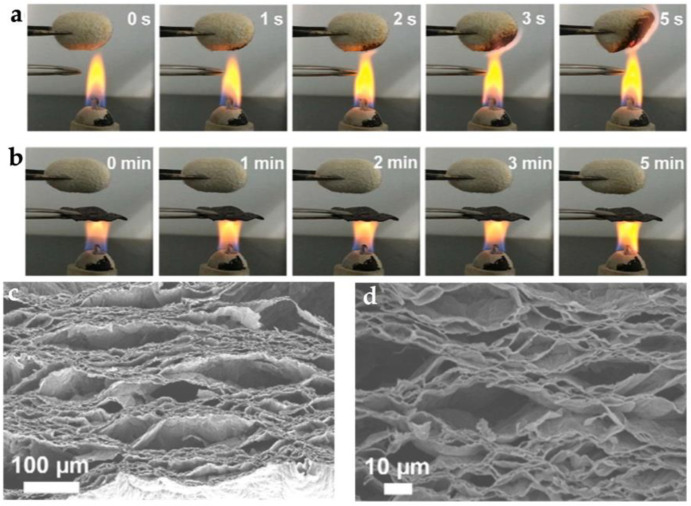
Fire test: (**a**) Combustion result of a silk cocoon; (**b**) Combustion results of silk cocoon protected by rGO-MMT-PVA; (**c**,**d**) SEM image of different sizes of rGO-MMT-PVA after flame treatment [86].

Peng et al. [86] used a vacuum-assisted filtration self-assembly process to achieve synergistic toughening of GO and MMT nanoplates and prepared a PO-MMT-PVA multifunctional ternary bio-inspired nanocomposite material. The synergistic toughening effect of graphene oxide and MMT nanoplates successfully realized the integration of strong and tough ternary bio-inspired nanocomposite materials, with a tensile strength of 356.0 MPa and toughness of 7.5 MJ/m^3^, which is superior to most binary graphene oxide-based layered materials. At the same time, the biomimetic nanocomposite material exhibits excellent flame retardancy. When used as a protective layer for silkworm cocoons, it can protect the cocoon from ignition for at least 5 min, as shown in Figure 22.

### 5.3. Responsive

**Figure 23:**
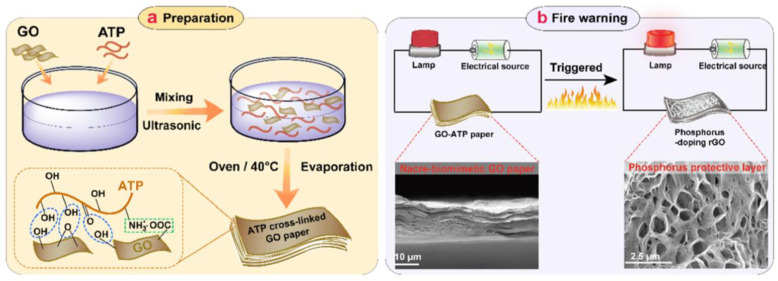
(**a**) Preparation of the GO-ATP papers; (**b**) mechanism of early fire warning [87].

Many responsive materials in BMOIs are highly sensitive to external environmental factors such as temperature and water, which is perfectly suited for the requirements of smart devices. Smart devices are primarily designed to automatically provide corresponding feedback based on changes in the external environment, achieving an intelligent purpose. This causes BMOIs to have great potential in the field of smart devices. Ma et al. [87] developed a fire warning sensor that can be used before combustion. They used a low-temperature evaporation assembly method, with GO and adenosine triphosphate (ATP) as the substrate, to prepare a “brick-and-mortar”-structure imitation pearl paper by utilizing hydrogen bonding and electrostatic attraction between GO and ATP molecules. Then, a fire alarm sensor was designed based on the high-temperature thermal reduction characteristics of graphene oxide, as shown in Figure 23. The material not only has a fast flame detection response (1.36 s) and a long continuous alarm time (1300 s) in paper containing 50 wt% ATP, but also exhibits flexibility, flame retardancy, thermal insulation performance, and charring performance.

**Figure 24:**
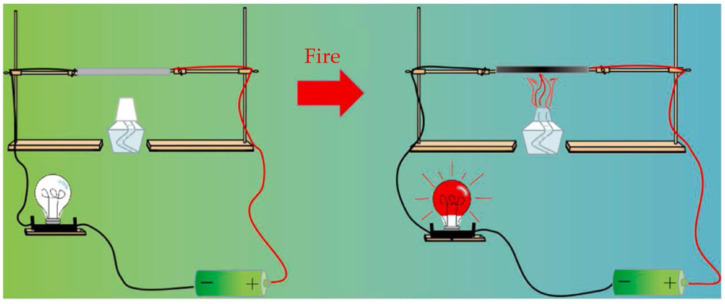
Fire-alarm schematic [88].

Yuan et al. [88] developed a boron-modified graphene oxide (GO-BA) paper for use in early fire alarm sensors using a green and easy-to-evaporate self-assembly method. The method has advantages such as simplicity, low cost, and environmental friendliness. The material uses GO as brick and boric acid (BA) as mortar to construct a “brick-and-mortar” structure through hydrogen bonds between them. Notably, the insulating GO-BA paper can rapidly thermally reduce to electrically conductive reduced graphene oxide (rGO) when exposed to flames, providing an ideal fire alarm response time of ~0.8 s. The alarm mechanism is shown in Figure 24. Furthermore, the oxidized boron formed under the flame attack covers the surface of the GO paper, suppressing further oxidation of the GO paper while insulating it from the external environment, thereby enhancing the material’s flame-retardant properties.

### 5.4. Electromagnetic Shielding

**Figure 25:**
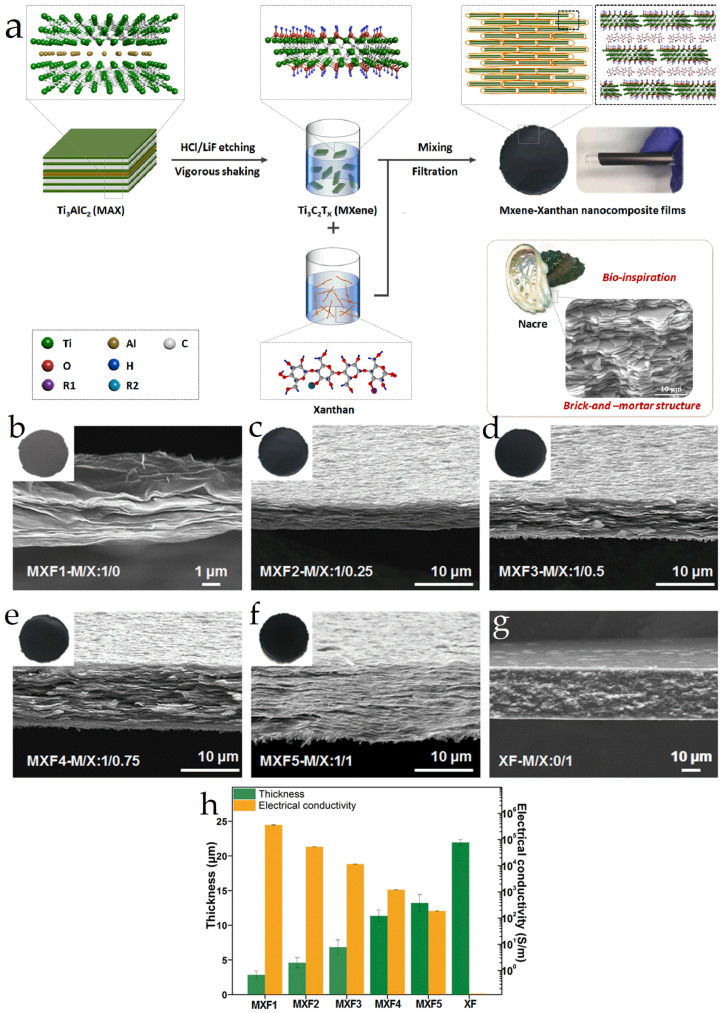
(**a**) Synthetic path graph of Ti_3_C_2_Tx MXene-xanthan nanocomposite films; (**b**–**f**) SEM images of MXene-xanthan nanocomposite films with different volume ratios of MXene and xanthan (MXF1-MXF5); (**g**) neat xanthan film; (**h**) thickness and electrical conductivity of MXF1-MXF5 [89].

BMOIs exhibit excellent electromagnetic shielding performance, which can effectively address the issue of mutual interference among electronic components caused by magnetic fields generated by electric currents. Sun and colleagues [89] successfully synthesized Ti_3_C_2_T_x_ MXene-xanthan nanocomposite films with outstanding electromagnetic shielding properties using vacuum-assisted filtration in a vacuum, and prepared MXene-xanthan nanocomposite films with varying volume ratios of MXene and xanthan (designated as MXF1-MXF5). The synthesis route is depicted in Figure 25a. The SEM images (Figure 25b–g) clearly show a layered structure, whereas the xanthan gum film lacks such a structure, indicating the formation of a highly organized “brick-and-mortar” structure. The electrical conductivity of the composite films was significantly improved compared to previous reports on MXene-based composite films (361,066 ± 2084–187 ± 6.6 S m^−1^ from MXF1 to MXF5), and the films also exhibited high tensile strength (up to 121.09 ± 7.96 MPa for MXF5), as demonstrated in Figure 25h.

### 5.5. Anti-Corrosion

**Figure 26:**
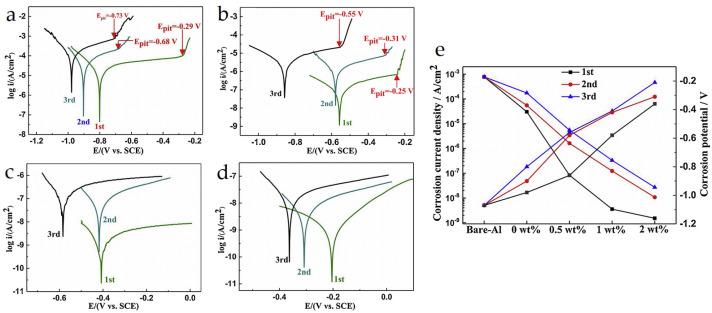
Tafel plots of (**a**) wSBRand B-GO/wSBRc with (**b**) 0.5 wt%, (**c**) 1 wt% and (**d**) 2 wt% GO pasted Al-2024 at 1st, 2nd and 3rd stages; (**e**) corrosion current density (icorr) and corrosion potential (Ecorr) [90].

Exposure to the external environment can cause corrosion and deteriorate the properties of materials over time, especially for materials that come into contact with corrosive liquids. The anti-corrosion materials in BMOIs can effectively prevent this problem and can be used as coatings for materials placed outdoors for a long time, containers that come into contact with corrosive liquids, and so on. Wang et al. [90] prepared a novel bioinspired material, B-GO/wSBRc, by using graphene oxide (GO) and waterborne styrene butadiene rubber (wSBR), which exhibited excellent anti-corrosion properties. As shown in Figure 26a–d, the Epit values of B-GO/wSBRc were reduced to a certain extent. The corrosion current density obtained by Tafel extrapolation method and corrosion potential (Figure 26e) clearly showed that with the increase in GO content, the Ecorr value gradually increased in the first (from −1.07 V/SCE to −0.21 V/SCE), second (from −1.07 V/SCE to −0.31 V/SCE) and third stages (from −1.07 V/SCE to −0.36 V/SCE). This indicates that layered graphene oxide can effectively enhance the anti-corrosion ability of wSBRc. The main mechanism for the enhancement of anti-corrosion includes: (1) B-GO/wSBRc cured at low temperature has no micro-pores, which can efficiently block the electrolyte from contacting the substrate; (2) GO can be well dispersed in wSBRc and maximize the diffusion path; and (3) π-π stacking between GO and wSBRc enhances their interaction.

### 5.6. Other

**Figure 27:**
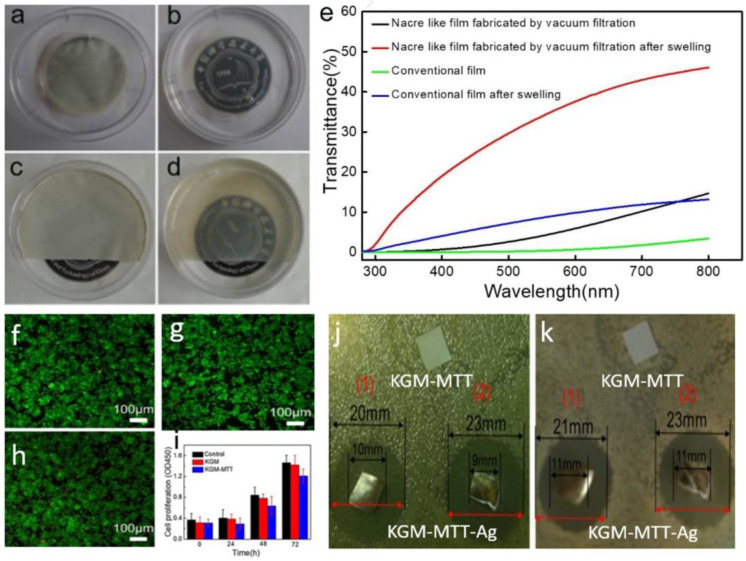
(**a**) Before and (**c**) after pictures of water absorption of the conventional KGM-MTT blend films; (**b**) Before and (**d**) after pictures of water absorption of the KGM-MTT; (**e**) UV/visible light transmittance test results; (**f**) micrographs of cocultured cells of control group, (**g**) KGM films, (**h**) KGM-MTT composite films; (**i**) proliferation of cells after co-culture; (**j**) antibacterial action against staphylococci; (**k**) antibacterial action against Escherichia coli, (1) 0.1 mol/L of silver ion concentration, (2) 0.2 mol/L of silver ion concentration [91].

Zhu et al. [91] prepared a biomimetic “brick-and-mortar”-structure composite film using vacuum filtration and konjac glucomannan–montmorillonite (KGM-MTT). The optical transparency and mechanical properties (tensile strength of 116 MPa) of the KGM-MTT composite film were significantly improved. The prepared composite film had a pearl-like “brick-and-mortar” structure with good transparency and mechanical properties. Under humid conditions, the pearl-like KGM-MTT film had a transparency of 20–50% in the visible spectrum, while the traditional KGM-MTT hybrid film had a transparency of 5–9%, as shown in Figure 27a–e. The ultimate tensile strength of the artificial pearl film was 2–3 times higher than that of films prepared by conventional methods. The cytotoxicity evaluation showed that the prepared KGM-MTT film was biocompatible, as shown in Figure 27f–i. Furthermore, this nacre-like KGM-MTT composite film can serve as a platform for introducing Ag nanoparticles, providing broad potential for antibacterial applications of such films, as shown in Figure 27j,k. In summary, KGM-MTT composite films impregnated with silver nanoparticles are very useful materials in food packaging and healthcare systems.

**Figure 28:**
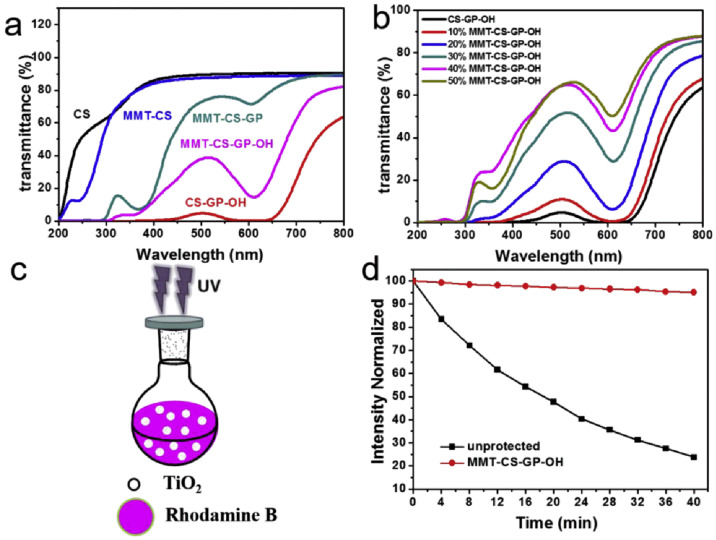
(**a**) UV-vis spectra of CS, CS-GP-OH, MMT-CS, MMT-CS-GP, and MMT-CS-GP-OH films; (**b**) UV-vis spectra of MMT-CS-GP-OH films with different MMT contents; (**c**) schematic illustration of UV-shielding performance testing of the MMT-CS-PG-OH film; (**d**) photocatalytic degradation profile of rhodamine B unprotected and protected by the MMT-CS-PG-OH film [92].

Liang et al. [92] successfully prepared a novel GP-reinforced MMT-CS-GP-OH composite material using a self-assembly process based on evaporation, with the use of GP (genipin), CS (chitosan), and MMT. They systematically studied the relationship between MMT content, CS-GP crosslinking, pH value, and the mechanical properties and interface interactions of the composite film. The results showed that the biologically inspired nano-composite material prepared had a synergistic effect originating from hydrogen bonds and covalent bonds, making its tensile strength 226 MPa and toughness 5.1 MJ/m^3^. Moreover, since GP is a natural crosslinker that can spontaneously react with CS to produce a deep blue pigment, the artificial pearl has excellent anti-UV universality (10.0% at 365 nm), as shown in Figure 28. Therefore, this integrated nacre-like MMT-CS nano-composite material is likely to be used in aerospace, biomedical and industrial fields in future.

**Figure 29:**
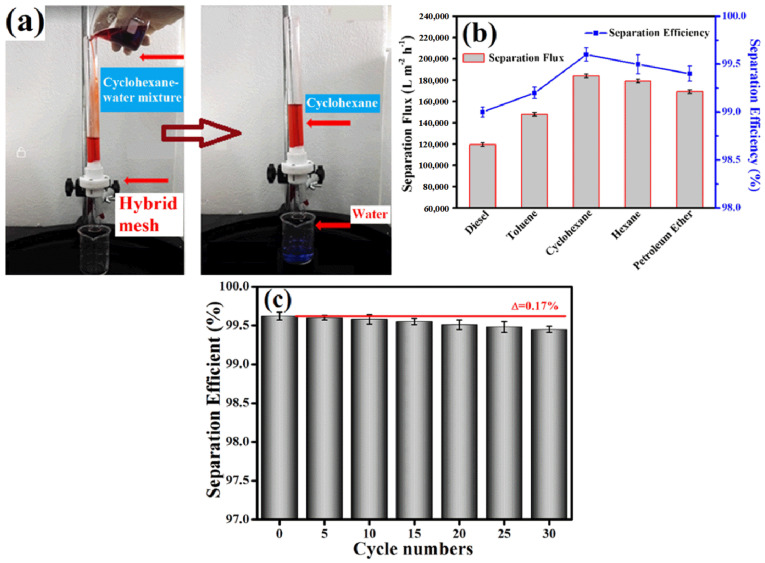
(**a**) Image of GO-CaCO_3_ oil–water separation; (**b**) separation flux and efficiency of the GO-CaCO_3_; (**c**) reusability of the GO-CaCO_3_ [93].

Dai et al. [93] prepared an underwater super-oleophobic hybrid mesh (GO-CaCO_3_) through LBL of GO and calcium carbonate (CaCO_3_). They verified its oil–water separation ability using a homemade oil–water separation device, as shown in Figure 29a. The solution in the figure is a mixture of cyclohexane and water, and is stained with Sudan III and methylene blue for observation. As seen in the figure, water can quickly pass through the hybrid mesh while cyclohexane cannot, achieving the purpose of oil–water separation. Additionally, by replacing cyclohexane with other oils and calculating the water flux and separation efficiency, the results in Figure 29b indicate that the hybrid mesh exhibits high separation efficiency and water flux far exceeding that of other studies for a certain oil–water mixture. Moreover, after multiple cycles of oil–water separation experiments, the hybrid mesh maintained excellent separation efficiency, as shown in Figure 29c. The hybrid mesh can be widely applied in the field of oily wastewater treatment.

**Figure 30:**
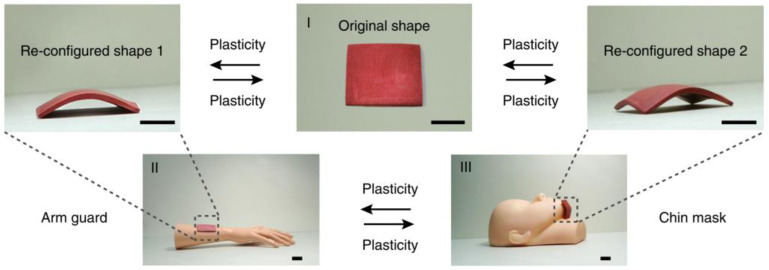
Shape re-configuration of CS-CaCO_3_ [95].

BMOIs have far-reaching applications beyond their current applications (Table 3). For instance, Chen et al. [94] developed a highly transparent and oil-repellent film using CS and CaCO_3_, which could be applied to swimming goggles and lenses. Du et al. [95] produced self-healing and shape-programmable structural materials using a thermally switchable Diels-Alder network polymer and alumina, which could meet the shape demands of different parts of the human body (Figure 30). Although BMOIs have been widely applied in various fields, the current research mainly focuses on their properties, and further exploration of their potential applications is needed.

## 6. Conclusions

One of the important components of composite materials is BMOIs, which greatly expand the application of composite materials. Inspired by the nacreous layer of pearls, BMOIs utilize organic substances as “mortar” and inorganic substances as “bricks”, which are connected in alternation to form a highly regular “brick-and-mortar” structure that provides BMOIs with exceptional mechanical and barrier properties. The diversity of raw materials and design of interfacial forces can make BMOIs multifunctional and meet the demands of various fields. Compared with conventional composite materials, BMOIs not only provide excellent mechanical properties such as high strength and toughness, but also offers UV resistance, flame retardancy, and corrosion resistance.

It is well known that the choice of raw materials has a significant impact in the performance of composite materials. The inorganic materials of BMOIs mainly include MMT and GO, which can provide the material with high mechanical properties and barrier properties, meeting the needs of high strength, toughness, fire resistance, and corrosion resistance. Additionally, GO exhibits excellent electrical properties and can also enable BMOI to have a place in materials fields such as capacitors and electrodes.

In the previous discussion, we detailed the interfacial interaction forces, including non-covalent bonds (hydrogen bonds, ionic bonds, and π-π interactions) and covalent bonds. Different interfacial forces can be designed based on different raw materials, with varying bond strengths. Generally, covalent bonds are more stable and suitable for materials requiring high strength, while adding other non-covalent bonds can further enhance the performance. In order to simplify production processes, yield, and other requirements, non-covalent bonds such as hydrogen bonds can be chosen. Therefore, BMOIs have high designability and can be adjusted according to design requirements.

The synthesis methods described earlier can all produce high-strength and high-toughness BMOIs, but each strategy has its advantages and disadvantages. FC allows for fine control of the thickness of layers and can simulate some of the nanoscale structural features of nacre, but it cannot provide a finely controlled platelet size distribution, especially at scales below 1μm. In contrast, LBL enables high-precision ordering of nanoscale building blocks, producing well-defined nanostructures. It not only allows for highly laminated and ultra-thin composite materials, but the close contact between layers typically results in high interfacial bonding and mechanical strength. However, due to its multi-step nature, the LBL technique is time and effort consuming. VF is energy efficient, but the synthesized materials often have low density and high porosity, resulting in poor mechanical properties.

As research on BMOIs continues to develop, its applications have been further expanded, not only in traditional material fields but also in smart materials, drug delivery, aerospace, and other areas. In the future, the high performance and designability of BMOIs will enable it to expand into even more fields.

Currently, there are still many common challenges in the research field of BMOIs. Firstly, different preparation methods have their own advantages and disadvantages. The LBL method is time consuming and labor intensive, the control accuracy of low-scale FC is insufficient, the mechanical performance of vacuum filtration is relatively weak, and the scale limitations of 3D printing technology are also problematic. Therefore, one of the main directions of future research is to optimize the design and synthesize an efficient, green, simple, high-precision, large-scale, and low-cost synthesis strategy. Secondly, there is insufficient research on functionalized BMOIs, and in the future, BMOIs should not only have excellent mechanical properties but also possess other properties such as self-healing and shape-changing capabilities to meet the requirements of a wider range of fields. Despite the many problems and challenges faced by BMOIs, they can be foreseen that it will be one of the development directions for high-performance, multifunctional materials.

## Figures and Tables

**Table 1:** List of raw materials.

	Function	Raw Material	Reference
Inorganic raw material	Brick	Montmorillonite	[35,36,37]
		Graphene oxide	[38,39,40]
		MXene	[41,42,43]
Organic raw material	Mortar	Polyvinyl alcohol	[44,45]
		Cellulose and its derivatives	[46,47,48]

**Table 2:** Advantages and disadvantages of strategies.

Strategies		Advantage	Disadvantage	Reference
Layer-by-layer assembly		High-precision, regular nanocomposites	Time consuming and labor intensive	[57]
Vacuum filtration		Energy efficient	Low density and high porosity	[59]
Freeze casting		Fine control at large size	Imprecise control in small size	[31,61,62,63,64]
Others	Co-extrusion	Large-scale processing	Imprecise control	[65,66,67]
	3D printing	Generate 3D complex structures	Small-scale production	[68,69]

**Table 3:** Properties and application.

Properties	Application	References
Light weight and high strength material	Structural material of construction, aviation, and biomedical	[81]
Flame-retardant materials	Fireproof materials of construction, electronics	[47,82,83,84,85,86]
Responsive materials	Inductor of smart fields	[87,88]
Electromagnetic shielding materials	Isolation layer electronic components	[89]
Anti-corrosion materials	Protective film of objects that come into contact with corrosive liquids	[90]
Other applications	Oil/water separation net/membrane	[91,92,93,94,95]

## Data Availability

Not applicable.
